# Healthcare Providers’ Advice for Tobacco Cessation and Its Association With Quit Attempts: A Cross-sectional Study in Japan, 2024

**DOI:** 10.2188/jea.JE20250277

**Published:** 2026-09-05

**Authors:** Satomi Odani, Takahiro Tabuchi

**Affiliations:** 1 The Tokyo Foundation for Policy Research, Tokyo, Japan; 2 Tohoku University Graduate School of Medicine, Sendai, Japan

**Keywords:** cessation, non-cigarette tobacco products, prevalence, primary health care, surveillance and monitoring

## Abstract

**Background:**

Despite guideline recommendations to deliver cessation advice, implementation in Japan remains inconsistent. We examined tobacco use prevalence, receipt of healthcare providers’ advice, and its association with quit attempts.

**Methods:**

We analyzed the 2024 wave of a nationwide web-based survey (*N* = 27,374; ages 16–74 years). Non-probability quota sampling and post-stratification weighting were used to approximate national population distributions. Current (past 30-day) tobacco use was assessed for heated tobacco products (HTPs), cigarettes, non-cigarette combustible tobacco, and dual use (HTPs plus combustible). Exposure was receipt of healthcare providers’ advice, and outcome was quit attempt in the past year. Associations were analyzed using multivariable Poisson regression to obtain adjusted prevalence ratios (APRs) and 95% confidence intervals (CIs).

**Results:**

Overall, 24.2% reported current any tobacco product use: 12.9% HTPs, 17.8% cigarettes, 3.7% non-cigarette combustible, and 7.3% dual use. Among any tobacco users (*N* = 6,361), 13.2% received advice and 26.7% attempted to quit. Among any tobacco users currently receiving medical care (*N* = 2,779), 20.9% received advice, primarily from doctors (10.3%) and nurses (8.0%), and 29.9% attempted to quit. Compared with no advice, quit attempts were more likely when advice came from one professional (APR 1.28; 95% CI, 1.05–1.58) or multiple professionals (APR 1.61; 95% CI, 1.17–2.23). Exclusive HTP users were less likely than exclusive combustible tobacco smokers to attempt quitting (APR 0.57; 95% CI, 0.41–0.81).

**Conclusion:**

Receipt of providers’ advice remains low in Japan yet is strongly associated with quit attempts, especially with multi-professional involvement. Policy, training, and system-level supports are needed to strengthen provider engagement.

## INTRODUCTION

Japan has a unique tobacco product use landscape, with heated tobacco products (HTPs) being the second most commonly used product, following traditional cigarettes.^1^^–^^3^ In 2023, the national prevalence of HTP use was 12.4%, compared to 18.9% for cigarettes.^1^ As the number of individuals using HTPs—either exclusively or alongside cigarettes—continues to rise, these products have become part of tobacco cessation services at healthcare facilities, which are covered by health insurance in the same way as cigarettes.^4^ Both the World Health Organization (WHO) and clinical guidelines encourage healthcare providers to actively screen for tobacco use and advise patients to quit as evidence-based strategies to promote both quit attempts and sustained cessation.^5^^–^^7^

However, experts have pointed out that these efforts are not universally implemented across Japan. The use of tobacco cessation outpatient services has constantly declined since 2014, following the introduction of HTPs,^8^ possibly because some smokers have turned to HTPs as a way to quit smoking.^9^ Additionally, since 2021, tobacco cessation outpatient services have been suspended at many healthcare facilities due to the global suspension of varenicline (Champix^®^),^10^ which is the only non-nicotine-containing oral medication approved for tobacco cessation in Japan, although other cessation aids are available.^11^ Reports indicate that an increasing number of individuals who wish to quit are unable to access appropriate medical support.^12^ These challenges emphasize the critical role of healthcare providers in guiding patients toward cessation in their routine clinical settings.

In light of the above, this paper aims to inform strategic public health policies and healthcare practices by examining the current implementation of cessation advice in Japan. Specifically, the objectives of this paper are: 1) to assess the most recent prevalence of tobacco product use in Japan, 2) to evaluate the extent to which individuals receive healthcare providers’ advice to quit, and 3) to analyze the relationship between receiving cessation advice and the likelihood of making a quit attempt.

## METHODS

### Data source

The present study conducted a cross-sectional analysis using data from the 2024 wave of the Japan Society and New Tobacco Internet Survey (JASTIS). This nationwide survey was administered online and relied on self-reported responses. Participants were recruited from the Rakuten Insight^13^ opt-in, non-probability online panel (>2 million members). To approximate the Japanese adult population, recruitment used quota sampling across key demographics (eg, age, sex, region, education, marital status, housing), consistent with national census categories. Data collection occurred between January 24 and February 27, 2024. To enhance data quality, 2,732 respondents were excluded due to implausible responses, such as selecting identical answers across multiple questions, choosing all options for inquiries related to illegal substance use or chronic conditions, or failing a validation question.^14^ Additionally, 1,894 individuals aged 75 years or older were excluded to maintain consistency with previous research.^1^^–^^3^ The final analytic sample comprised 27,374 participants. Further details on the sampling methodology are available elsewhere.^14^ The study was approved by the Research Ethics Committee of the Osaka International Cancer Institute (approval number 20094-2). The data that support the findings of this study are available from the corresponding author upon reasonable request.

### Measures

#### Prevalence of current tobacco product use

Current tobacco product use was defined based on self-reported consumption within the past 30 days. The survey assessed the use of various tobacco products, including HTPs, manufactured and roll-your-own cigarettes, and other (non-cigarette) combustible tobacco products such as little cigars, pipes, and water pipes. Dual use of HTPs and combustible tobacco was also examined. Participants who reported using a specific product on at least one day in the past 30 days were classified as current users of that product. To enhance clarity and response accuracy, the questionnaire listed product names alongside designated response fields for each tobacco product. Additionally, well-known HTP brands (eg, Ploom Tech, Ploom S, Ploom X, IQOS, glo, and lil HYBRID) were provided as examples to help participants accurately identify the products they had used.

#### Receipt of cessation advice from healthcare providers

Respondents were asked whether they had received tobacco cessation advice from healthcare providers in the past year and to specify which professionals provided the advice, including doctors, nurses, pharmacists, dentists, and other healthcare providers. In addition to calculating the overall percentage of current tobacco users who received cessation advice and the breakdown by healthcare professional, we further assessed the intensity of the advice received. This was done by categorizing respondents based on the number of healthcare professionals involved: no advice, advice from one professional, or advice from two or more professionals.

#### Quit attempts

Quit attempts were assessed with the question, “*During the past year, did you stop using tobacco for one day or longer because you were trying to quit?*”

#### Covariates

The following covariates were considered: sex, age, educational attainment, marital status, urbanization, alcohol use, and whether respondents were currently receiving medical care. Urbanization was defined based on Densely Inhabited District data from the 2020 Population Census of Japan.^15^^,^^16^ Current receipt of medical care was used as a proxy indicator for recent healthcare visits, assessed by asking respondents whether they were currently receiving treatment for any physical or mental health conditions. Additionally, tobacco use status was classified into three categories for current users: exclusive combustible tobacco use, exclusive HTP use, and dual use.

### Statistical analysis

Descriptive analyses were performed to assess the prevalence of tobacco product use, as well as the proportion of current users who received cessation advice from healthcare providers and attempted to quit. To examine the association between receiving cessation advice and making a quit attempt, multivariable Poisson regression was applied. Adjusted prevalence ratios (APRs) with 95% confidence intervals (CIs) were estimated, controlling for all previously mentioned covariates. To account for potential biases due to the internet-based sampling, all analyses were weighted using inverse probability weighting (IPW). Propensity scores for being an internet survey respondent were derived using logistic regression models, which were adjusted for key demographic, socioeconomic, health-related, and tobacco-use factors, comparing the 2024 JASTIS sample with a nationally representative sample.^17^ Detailed information on the weighting procedure is available in previous publications.^14^^,^^18^ Statistical analyses were conducted using R version 4.1.3 (R Foundation for Statistical Computing, Vienna, Austria).

### Sensitivity analysis

Because temporal ordering is uncertain in this cross-sectional survey and quit motivation may lie on either side of the exposure (as pre-exposure readiness to quit or as a post-exposure mediator influenced by advice), we did not adjust for motivation in the primary models. As a sensitivity (subgroup) analysis, we stratified tobacco users currently receiving medical care by quit motivation (yes/no) and re-estimated the multivariable Poisson regression models within each stratum using the same covariate set and weighting procedure to assess robustness. Quit motivation was defined from product-specific items asking whether respondents were interested in quitting HTPs or combustible tobacco within 1 month/6 months/sometime (coded yes) versus not interested (coded no); for dual users, motivation was coded yes if interest was endorsed for either product.

## RESULTS

A total of 27,374 individuals aged 16–74 years were included. Respondent distributions (unweighted counts and weighted percentages) for the overall sample, tobacco users, and tobacco users currently receiving medical care are presented in eTable 1.

### Prevalence of current tobacco product use

Overall, the prevalence of current (past 30-day) tobacco product use was 12.9% for heated tobacco products (HTPs), 17.8% for cigarettes, and 3.7% for non-cigarette combustible tobacco (Table 1). Additionally, 7.3% of respondents reported concurrent use of HTPs and combustible tobacco. Overall, 24.2% of respondents were current users of any tobacco product. Notably, even among respondents below Japan’s legal age for tobacco use (ie, <20 years), 7.3% reported current tobacco use. Tobacco use prevalence varied significantly by demographic and behavioral characteristics. The prevalence of current tobacco product use was approximately 2.5 times higher among men than women (34.9% vs 14.0%) and nearly twice as high among current drinkers compared to non-drinkers or those who had never consumed alcohol (31.0% vs 16.5%). Additionally, individuals with lower educational attainment (high school or below) had a roughly 5-percentage-point higher prevalence of tobacco use than those with education beyond high school (26.8% vs 21.7%). Similarly, tobacco use prevalence was higher among individuals currently receiving medical care than those who were not (27.0% vs 22.5%).

**Table 1.** Prevalence of current (past 30-day) tobacco product use in Japan, 2024

Characteristics	Heated tobacco products	Cigarettes	Non-cigarette combustible tobacco products	Dual (combustible + heated tobacco) use	Any tobacco product

	Weighted % (95% CI)	Weighted % (95% CI)	Weighted % (95% CI)	Weighted % (95% CI)	Weighted % (95% CI)
Total	12.9 (12.2–13.6)	17.8 (17.1–18.6)	3.7 (3.4–4.1)	7.3 (6.8–7.8)	24.2 (23.4–25.1)
Sex					
Female	7.0 (6.3–7.7)	9.9 (9.1–10.6)	1.9 (1.6–2.2)	3.5 (3.1–4.0)	14.0 (13.0–14.9)
Male	19.1 (18.0–20.3)	26.1 (24.9–27.4)	5.7 (5.0–6.3)	11.2 (10.3–12.1)	34.9 (33.5–36.3)
Age, years					
16–19	6.2 (1.1–11.2)	4.7 (0.5–8.8)	4.2 (0.1–8.3)	3.9 (0.2–7.6)	7.3 (2.2–12.4)
20–29	16.2 (14.4–18.1)	15.6 (13.9–17.4)	7.3 (6.1–8.4)	11.4 (9.8–12.9)	21.9 (19.9–24.0)
30–39	16.9 (15.1–18.8)	18.7 (16.8–20.6)	4.5 (3.6–5.4)	10.1 (8.6–11.6)	26.5 (24.4–28.6)
40–49	17.1 (15.4–18.9)	20.9 (19.1–22.7)	3.1 (2.4–3.8)	8.6 (7.4–9.9)	30.1 (28.0–32.1)
50–59	12.1 (10.8–13.5)	19.6 (17.9–21.3)	2.0 (1.4–2.5)	5.5 (4.5–6.4)	26.9 (25.0–28.7)
≥60	6.0 (5.1–7.0)	16.4 (15.0–17.8)	2.3 (1.7–2.9)	3.0 (2.4–3.7)	19.8 (18.3–21.3)
Education					
High school or below	14.2 (13.1–15.4)	19.7 (18.4–20.9)	3.5 (2.9–4.0)	7.8 (6.9–8.7)	26.8 (25.4–28.2)
Beyond high school	11.7 (11.0–12.3)	16.0 (15.3–16.7)	4.0 (3.7–4.4)	6.8 (6.3–7.3)	21.7 (20.9–22.5)
Marital status					
Single	12.6 (11.4–13.9)	16.2 (14.9–17.4)	5.1 (4.3–5.9)	8.2 (7.1–9.2)	21.7 (20.2–23.1)
Married/cohabiting	13.3 (12.4–14.2)	18.4 (17.4–19.4)	3.3 (2.9–3.7)	7.1 (6.5–7.8)	25.2 (24.1–26.3)
Separated/divorced/widowed	11.1 (9.3–13.0)	19.4 (16.8–21.9)	2.4 (1.7–3.1)	5.4 (4.1–6.7)	25.8 (23.0–28.5)
Urbanization					
Urban (DID)	13.0 (12.2–13.8)	17.3 (16.5–18.2)	4.0 (3.6–4.5)	7.1 (6.5–7.7)	23.9 (22.9–24.9)
Rural (non-DID)	12.8 (11.6–14.1)	18.6 (17.2–20.0)	3.3 (2.7–3.9)	7.6 (6.6–8.5)	24.7 (23.2–26.3)
Alcohol use					
Non-current/never	8.4 (7.6–9.2)	11.8 (10.8–12.7)	2.0 (1.6–2.4)	4.2 (3.6–4.8)	16.5 (15.4–17.6)
Current	16.9 (15.9–17.9)	23.2 (22.1–24.3)	5.3 (4.7–5.9)	10.0 (9.2–10.8)	31.0 (29.8–32.3)
Currently receiving medical care					
No	12.5 (11.6–13.3)	15.9 (15.0–16.9)	2.8 (2.4–3.1)	6.7 (6.1–7.4)	22.5 (21.5–23.6)
Yes	13.7 (12.5–14.8)	20.8 (19.5–22.1)	5.3 (4.6–6.0)	8.2 (7.3–9.0)	27.0 (25.5–28.4)

CI, confidence interval; DID, Densely Inhabited District.

Note: A total of 27,374 individuals were included. Data were weighted to address the selectivity of the internet-based sample using a nationally representative sample of Japanese population as the reference. “Currently receiving medical care” serves as a proxy for recent visits to hospitals, clinics, or dental clinics. Cigarettes assessed in this study include manufactured cigarettes and roll-your-own cigarettes. Non-cigarette combustible tobacco products include little cigars, pipes, and water pipes. Heated tobacco products include Ploom Tech, Ploom S, Ploom X, IQOS, glo, and lil HYBRID.

### Receipt of healthcare providers’ advice for cessation

Among current users of any tobacco product (*N* = 6,361), 13.2% reported receiving tobacco cessation advice from a healthcare provider. Specifically, 6.8% received advice from doctors, 4.7% from nurses, 1.5% from pharmacists, 1.2% from dentists, and 0.9% from other medical professionals (Table 2). Among those currently receiving medical care for physical or mental conditions (*N* = 2,779), 20.9% reported receiving tobacco cessation advice. This included 10.3% who received advice from doctors, 8.0% from nurses, 3.1% from pharmacists, 2.1% from dentists, and 1.5% from other medical professionals. The receipt of cessation advice varied across demographic characteristics. By sex, a higher percentage of men than women reported receiving advice (22.7% vs 15.8%). By tobacco use status, 38.6% of dual users of HTPs and combustible tobacco products reported receiving cessation advice, compared to 13.3% of exclusive combustible tobacco smokers and 12.8% of exclusive HTP users.

**Table 2.** Receipt of healthcare providers’ advice for cessation among current tobacco users in Japan, 2024

Tobacco product users, overall (*n* = 6,361)						

Characteristics	Total (any healthcare provider)	Doctors	Nurses	Pharmacists	Dentists	Other medical professionals

	Weighted % (95% CI)	Weighted % (95% CI)	Weighted % (95% CI)	Weighted % (95% CI)	Weighted % (95% CI)	Weighted % (95% CI)
Total	13.2 (11.9–14.5)	6.8 (5.9–7.7)	4.7 (3.9–5.5)	1.5 (0.9–2.1)	1.2 (0.8–1.6)	0.9 (0.6–1.3)
Tobacco product type						
Combustible tobacco only	8.6 (7.0–10.1)	5.8 (4.5–7.0)	2.1 (1.3–2.8)	0.4 (0.1–0.6)	0.9 (0.3–1.4)	0.3 (0.1–0.6)
HTP only	10.1 (7.4–12.8)	5.7 (3.8–7.6)	3.3 (1.4–5.1)	0.3 (0.1–0.5)	0.3 (0.0–0.7)	0.4 (0.1–0.8)
Dual use	22.9 (19.9–25.9)	9.2 (7.3–11.1)	9.9 (7.8–11.9)	4.2 (2.4–6.1)	2.3 (1.3–3.3)	2.2 (1.3–3.1)
Sex						
Female	10.1 (7.8–12.3)	4.5 (3.2–5.9)	3.3 (1.7–5.0)	0.9 (0.4–1.3)	1.0 (0.5–1.6)	1.0 (0.4–1.5)
Male	14.5 (12.9–16.2)	7.7 (6.5–8.9)	5.3 (4.3–6.3)	1.8 (1.0–2.6)	1.2 (0.7–1.7)	0.9 (0.5–1.3)
Age, years						
16–19	36.8 (1.2–72.4)	1.2 (0.1–2.3)	3.4 (0.7–6.2)	29.8 (1.0–58.6)	0.2 (0.0–0.4)	3.8 (0.2–7.4)
20–29	17.5 (14.4–20.7)	7.1 (5.0–9.2)	7.5 (5.4–9.5)	3.2 (1.6–4.8)	2.8 (1.3–4.4)	2.3 (0.9–3.6)
30–39	12.5 (9.5–15.4)	5.3 (3.2–7.3)	6.0 (3.8–8.1)	0.8 (0.3–1.3)	0.4 (0.1–0.8)	1.1 (0.4–1.8)
40–49	10.5 (8.1–12.9)	4.6 (2.8–6.3)	4.5 (2.8–6.2)	0.9 (0.3–1.6)	1.3 (0.5–2.1)	0.7 (0.1–1.3)
50–59	9.9 (7.5–12.3)	5.5 (4.0–7.0)	2.6 (1.5–3.7)	1.0 (0.1–2.0)	0.2 (0.0–0.5)	0.4 (0.0–0.9)
≥60	15.7 (12.3–19.1)	11.5 (8.8–14.2)	3.5 (1.3–5.6)	1.2 (0.2–2.2)	1.2 (0.3–2.2)	0.3 (0.0–0.7)
Education						
High school or below	12.0 (10.0–13.9)	6.6 (5.2–8.0)	4.3 (3.0–5.5)	1.1 (0.3–1.8)	1.1 (0.5–1.7)	0.5 (0.1–0.9)
Beyond high school	14.7 (13.3–16.2)	7.1 (6.1–8.1)	5.4 (4.4–6.4)	1.8 (1.3–2.3)	1.3 (0.9–1.7)	1.5 (1.0–2.1)
Marital status						
Single	12.7 (10.4–15.0)	6.2 (4.6–7.7)	4.7 (3.2–6.1)	2.1 (0.7–3.4)	1.3 (0.5–2.1)	1.3 (0.4–2.2)
Married/cohabiting	13.9 (12.2–15.7)	7.1 (5.8–8.3)	5.1 (4.0–6.3)	1.3 (0.6–2.0)	1.3 (0.7–1.8)	0.9 (0.5–1.2)
Separated/divorced/widowed	9.8 (6.5–13.0)	6.5 (3.9–9.0)	1.9 (0.6–3.2)	1.4 (0.4–2.4)	0.1 (0.0–0.3)	0.3 (0.1–0.6)
Urbanization						
Urban (DID)	13.6 (12.0–15.2)	6.9 (5.8–8.0)	4.7 (3.8–5.7)	1.8 (1.0–2.6)	1.3 (0.8–1.8)	1.0 (0.5–1.4)
Rural (non-DID)	12.7 (10.4–15.0)	6.6 (5.0–8.3)	4.7 (3.2–6.2)	1.1 (0.4–1.8)	1.0 (0.4–1.7)	0.9 (0.4–1.4)
Alcohol use						
Non-current/never	9.5 (7.5–11.6)	5.5 (4.0–6.9)	2.8 (1.4–4.2)	0.6 (0.3–0.9)	0.5 (0.1–0.9)	0.5 (0.2–0.8)
Current	15.0 (13.3–16.6)	7.4 (6.2–8.6)	5.6 (4.5–6.6)	1.9 (1.1–2.8)	1.5 (1.0–2.0)	1.1 (0.7–1.6)
Currently receiving medical care						
No	7.4 (6.2–8.7)	4.1 (3.1–5.1)	2.2 (1.5–2.9)	0.3 (0.1–0.5)	0.4 (0.2–0.7)	0.5 (0.2–0.7)
Yes	20.9 (18.4–23.3)	10.3 (8.6–12.1)	8.0 (6.3–9.7)	3.1 (1.8–4.4)	2.1 (1.3–3.0)	1.5 (0.9–2.2)

Tobacco product users, currently receiving medical care (*n* = 2,779)						

Characteristics	Total (any healthcare provider)	Doctors	Nurses	Pharmacists	Dentists	Other medical professionals

	Weighted % (95% CI)	Weighted % (95% CI)	Weighted % (95% CI)	Weighted % (95% CI)	Weighted % (95% CI)	Weighted % (95% CI)

Total	20.9 (18.4–23.3)	10.3 (8.6–12.1)	8.0 (6.3–9.7)	3.1 (1.8–4.4)	2.1 (1.3–3.0)	1.5 (0.9–2.2)
Tobacco product type						
Combustible tobacco only	13.3 (10.5–16.0)	9.1 (6.8–11.4)	3.3 (1.9–4.8)	0.6 (0.2–1.1)	1.6 (0.5–2.6)	0.5 (0.1–0.8)
HTP only	12.8 (7.6–17.9)	6.7 (3.8–9.5)	5.3 (0.9–9.8)	0.2 (0.0–0.5)	0.6 (0.1–1.1)	0.7 (0.2–1.3)
Dual use	38.6 (33.3–43.8)	14.8 (11.2–18.5)	17.3 (13.4–21.3)	9.1 (5.1–13.0)	4.0 (2.0–6.0)	3.8 (1.9–5.8)
Sex						
Female	15.8 (11.4–20.3)	6.9 (4.4–9.4)	7.0 (3.2–10.9)	1.6 (0.7–2.4)	1.4 (0.6–2.2)	1.4 (0.3–2.4)
Male	22.7 (19.7–25.7)	11.6 (9.5–13.8)	8.3 (6.5–10.2)	3.7 (2.0–5.4)	2.4 (1.3–3.5)	1.6 (0.8–2.4)
Age, years						
16–19	39.2 (2.3–76.1)	0.9 (0.0–1.8)	3.7 (0.3–7.2)	36.0 (0.9–71.1)	0.2 (0.0–0.5)	1.5 (0.2–2.8)
20–29	37.8 (29.6–46.1)	15.5 (9.9–21.0)	18.7 (12.8–24.7)	7.6 (3.3–11.9)	5.9 (1.9–9.9)	4.4 (0.7–8.1)
30–39	24.8 (18.0–31.6)	9.1 (5.1–13.1)	14.9 (8.8–21.1)	2.1 (0.7–3.4)	1.4 (0.2–2.6)	2.4 (0.7–4.2)
40–49	18.1 (13.0–23.2)	6.9 (3.3–10.6)	7.7 (4.3–11.2)	2.5 (0.8–4.3)	2.7 (0.7–4.7)	1.7 (0.1–3.3)
50–59	12.9 (8.5–17.3)	6.7 (4.4–9.1)	2.5 (0.9–4.0)	2.0 (0.0–4.0)	0.5 (0.0–1.1)	0.7 (0.2–1.2)
≥60	18.4 (13.9–23.0)	13.2 (9.7–16.6)	4.7 (1.6–7.7)	1.8 (0.2–3.4)	1.7 (0.3–3.1)	0.5 (0.1–1.0)
Education						
High school or below	18.9 (15.2–22.6)	9.9 (7.3–12.5)	7.2 (4.7–9.8)	2.4 (0.7–4.1)	2.0 (0.7–3.2)	0.9 (0.1–1.7)
Beyond high school	23.1 (20.5–25.8)	11.2 (9.4–13.1)	9.3 (7.4–11.3)	3.3 (2.3–4.2)	2.4 (1.4–3.4)	2.5 (1.4–3.7)
Marital status						
Single	26.3 (20.7–31.8)	12.2 (8.5–15.9)	10.8 (6.9–14.6)	4.9 (1.3–8.6)	2.9 (0.6–5.1)	2.5 (0.2–4.7)
Married/cohabiting	20.8 (17.6–23.9)	10.3 (8.1–12.4)	8.0 (5.8–10.2)	2.6 (1.1–4.1)	2.2 (1.2–3.2)	1.4 (0.7–2.1)
Separated/divorced/widowed	11.4 (7.1–15.6)	7.4 (3.7–11.1)	2.8 (0.9–4.7)	2.7 (0.7–4.6)	0.2 (0.0–0.4)	0.5 (0.0–1.1)
Urbanization						
Urban (DID)	20.7 (17.8–23.6)	10.1 (8.2–12.1)	8.2 (6.3–10.1)	3.6 (1.8–5.4)	2.0 (1.0–3.0)	1.5 (0.6–2.3)
Rural (non-DID)	21.0 (16.6–25.5)	10.7 (7.6–13.8)	7.7 (4.6–10.9)	2.4 (0.7–4.0)	2.3 (0.8–3.7)	1.6 (0.6–2.7)
Alcohol use						
Non-current/never	15.1 (10.9–19.3)	8.9 (5.9–12.0)	5.2 (2.0–8.3)	0.8 (0.4–1.3)	1.0 (0.1–1.9)	0.7 (0.1–1.2)
Current	23.3 (20.3–26.3)	11.0 (8.9–13.0)	9.2 (7.1–11.2)	4.1 (2.3–5.9)	2.6 (1.5–3.7)	1.9 (1.0–2.8)

CI, confidence interval; DID, Densely Inhabited District; HTP, heated tobacco product.

Note: Receipt of healthcare providers’ advice was assessed over the past year. Data were weighted to address the selectivity of the internet-based sample using a nationally representative sample of Japanese population as the reference. “Currently receiving medical care” serves as a proxy for recent visits to hospitals, clinics, or dental clinics. Cigarettes assessed in this study include manufactured cigarettes and roll-your-own cigarettes. Non-cigarette combustible tobacco products include little cigars, pipes, and water pipes. Heated tobacco products include Ploom Tech, Ploom S, Ploom X, IQOS, glo, and lil HYBRID.

### Quit attempts

Among current tobacco users, 26.7% reported a quit attempt in the past year (Table 3). Quit attempts were significantly more common among those who received cessation advice from healthcare providers: compared with no advice, advice from one professional was associated with a 25% higher likelihood of attempting to quit (APR 1.25; 95% CI, 1.06–1.47), and advice from two or more professionals with a 55% higher likelihood (APR 1.55; 95% CI, 1.17–2.04). By product type, individuals who exclusively used HTPs were significantly less likely to attempt quitting than exclusive combustible smokers (APR 0.67; 95% CI, 0.55–0.82). Quit attempts also declined with age, with the lowest likelihood observed among those aged 50–59 years versus 20–29 years (APR 0.42; 95% CI, 0.33–0.52). By educational attainment, those with a high school education or lower were less likely to attempt quitting than those with higher education (APR 0.83; 95% CI, 0.74–0.94). Finally, individuals currently receiving medical treatment for physical or mental conditions had a 19% higher likelihood of attempting to quit than those not receiving treatment (APR 1.19; 95% CI, 1.04–1.37).

**Table 3.** Quit attempts association with receipt of cessation advice from healthcare providers in Japan, 2024

Characteristics	Tobacco users, overall (*n* = 6,361)		Tobacco users, currently receiving medical care (*n* = 2,779)	

	Weighted % (95% CI)	APR (95% CI)	Weighted % (95% CI)	APR (95% CI)
Total	26.7 (24.9–28.5)	—	29.9 (27.1–32.7)	—
Receipt of cessation advice				
No advice	24.7 (22.9–26.6)	Ref.	26.4 (23.3–29.4)	Ref.
Yes (from one professional)	38.5 (32.6–44.3)	1.25 (1.06–1.47)	41.3 (33.8–48.8)	1.28 (1.05–1.58)
Yes (from two or more professionals)	54.1 (41.4–66.7)	1.55 (1.17–2.04)	58.2 (43.8–72.6)	1.61 (1.17–2.23)
Tobacco product type				
Combustible tobacco only	25.5 (23.1–27.9)	Ref.	29.2 (25.3–33.1)	Ref.
HTP only	17.8 (14.6–21.1)	0.67 (0.55–0.82)	17.5 (11.5–23.5)	0.57 (0.41–0.81)
Dual use	35.4 (31.8–38.9)	1.13 (0.98–1.31)	39.3 (34.1–44.5)	1.04 (0.85–1.27)
Sex				
Female	28.8 (25.5–32.0)	Ref.	29.7 (24.3–35.1)	Ref.
Male	25.8 (23.7–27.9)	0.89 (0.78–1.02)	30.0 (26.7–33.3)	0.99 (0.81–1.20)
Age, years				
16–19	59.2 (23.9–94.5)	1.32 (0.85–2.04)	57.0 (14.6–99.4)	1.33 (0.79–2.24)
20–29	43.8 (38.5–49.0)	Ref.	45.8 (36.5–55.1)	Ref.
30–39	25.7 (21.8–29.6)	0.59 (0.48–0.72)	37.9 (29.7–46.1)	0.84 (0.63–1.14)
40–49	22.2 (18.9–25.4)	0.52 (0.42–0.63)	25.4 (20.0–30.9)	0.62 (0.45–0.85)
50–59	17.8 (14.8–20.8)	0.41 (0.33–0.52)	20.4 (15.9–25.0)	0.50 (0.36–0.71)
≥60	26.0 (22.2–29.8)	0.57 (0.46–0.70)	27.8 (22.8–32.8)	0.66 (0.49–0.90)
Education				
High school or below	24.5 (21.8–27.1)	0.83 (0.74–0.94)	28.2 (24.0–32.5)	0.90 (0.76–1.07)
Beyond high school	30.4 (28.4–32.3)	Ref.	33.0 (30.0–36.0)	Ref.
Marital status				
Single	30.2 (26.6–33.7)	0.88 (0.75–1.03)	36.7 (30.2–43.2)	1.00 (0.80–1.25)
Married/cohabiting	25.8 (23.7–28.0)	Ref.	28.2 (24.9–31.5)	Ref.
Separated/divorced/widowed	22.6 (17.0–28.2)	0.94 (0.73–1.20)	27.8 (19.0–36.5)	1.07 (0.78–1.47)
Urbanization				
Urban (DID)	27.5 (25.3–29.6)	Ref.	31.0 (27.5–34.5)	Ref.
Rural (non-DID)	25.5 (22.5–28.5)	0.95 (0.83–1.08)	28.3 (23.7–32.9)	0.93 (0.76–1.12)
Alcohol use				
Non-current/never	25.3 (22.1–28.5)	Ref.	25.5 (20.6–30.5)	Ref.
Current	27.3 (25.2–29.4)	1.04 (0.91–1.20)	31.8 (28.4–35.2)	1.15 (0.93–1.42)
Currently receiving medical care				
No	24.3 (22.0–26.5)	Ref.	—	—
Yes	29.9 (27.1–32.7)	1.19 (1.04–1.37)	—	—

APR, adjusted prevalence ratio; CI, confidence interval; DID, Densely Inhabited District; HTP, heated tobacco product; Ref., reference.

Note: Receipt of healthcare providers’ advice and quit attempts were both assessed over the past year. Data were weighted to address the selectivity of the internet-based sample using a nationally representative sample of Japanese population as the reference. “Currently receiving medical care” serves as a proxy for recent visits to hospitals, clinics, or dental clinics. Combustible tobacco products include cigarettes, roll-your-own cigarettes, little cigars, pipes, and water pipes. Heated tobacco products (HTPs) include Ploom Tech, Ploom S, Ploom X, IQOS, glo, and lil HYBRID.

When restricting the analysis to tobacco users receiving medical treatment, 29.9% reported making a quit attempt in the past year. The pattern of associations remained consistent with the overall analysis, except that educational attainment was not significantly associated with quit attempts in this subgroup. Those who received cessation advice from healthcare providers were 28% (APR 1.28; 95% CI, 1.05–1.58) and 61% (APR 1.61; 95% CI, 1.17–2.23) more likely to attempt quitting when advised by one and two or more professionals, respectively. Notably, exclusive HTP users were less likely than exclusive combustible smokers to attempt quitting (APR 0.57; 95% CI, 0.41–0.81). In sensitivity analyses stratified by quit motivation, the positive association between receipt of advice and quit attempts persisted in both strata (eTable 2). Without quit motivation, 12.8% reported a past-year quit attempt; compared with no advice, those who received advice from two or more professionals were significantly more likely to attempt quitting (APR 2.36; 95% CI, 1.12–4.98). With quit motivation, 35.9% reported a quit attempt; the likelihood was higher among those who received advice from one professional (APR 1.29; 95% CI, 1.05–1.60) and from two or more professionals (APR 1.48; 95% CI, 1.08–2.01).

## DISCUSSION

In 2024, 24.2% of respondents reported current tobacco product use. Cigarettes were the most commonly used product, with a prevalence of 17.8%, followed by HTPs at 12.9%. Dual use of combustibles and HTPs was 7.3%, implying that a majority of HTP users are dual users—consistent with a synthesis of 26 studies reporting that roughly two-thirds of HTP users co-use cigarettes.^19^ These figures have remained stable in recent years in Japan, showing no signs of decline.^1^^–^^3^ Among current users of any tobacco product, only 13.2% reported receiving cessation advice from healthcare providers. Even among those who were receiving medical care, just 20.9% reported receiving cessation advice. While the majority of tobacco users did not receive such advice, receipt of advice was significantly associated with increased quit attempts, particularly when users were advised by multiple healthcare professionals. Notably, the advice–quit attempt association persisted in both subgroups stratified by quit motivation (among those receiving medical care). These findings highlight the need to strengthen the implementation of tobacco cessation policies, particularly those that encourage the provision of cessation advice by all types of healthcare providers.

The percentage of tobacco users who received cessation advice from healthcare providers was low, with merely one in five tobacco users currently receiving medical care. This was lower compared to rates reported in other countries or regions such as the United States (50.5%),^20^ England (47.2%),^21^ Taiwan (72.3%),^22^ and China (65.4%).^23^ Moreover, the percentage declined compared to our previously reported figure from the 2020 wave of the same internet survey (from 33.0% to 22.7% for men and 23.9% to 15.8% for women, respectively),^24^ potentially attributed to the nationwide decrease in the utilization of cessation services following the suspension of varenicline in 2021.^8^^,^^10^

In Japan, tobacco use is routinely screened during medical visits and recorded in the medical system; however, healthcare providers should go beyond merely identifying tobacco users. As recommended by clinical guidelines,^6^^,^^7^ they should assess patients’ level of tobacco dependence, determine their readiness to quit, and provide appropriate advice, assistance, and referrals to cessation services. Several factors may contribute to the low rates of cessation counseling, including a lack of skills, low self-efficacy, limited referral resources, time constraints, and the perception that smoking cessation care falls outside healthcare providers’ responsibilities.^25^^–^^27^ However, research indicates that even brief interventions lasting just a few minutes during routine visits can effectively promote cessation, making them a practical approach in time-constrained settings.^28^ While Japan has established guidelines for smoking cessation treatment that include structured approaches to counseling, further integration of standardized and concise strategies may help enhance the consistency and effectiveness of cessation counseling. For instance, system-level interventions that raise healthcare providers’ awareness and provide training on the internationally recognized “5 A’s” model—Ask, Advise, Assess, Assist, and Arrange^29^—can help them confidently incorporate smoking cessation support into their practice.

Despite the limited delivery of cessation advice from healthcare providers, receiving such advice was positively associated with making a quit attempt, with a stronger effect observed when patients were advised by multiple medical professionals. This finding aligns with previous studies demonstrating the beneficial impact of healthcare providers’ advice on tobacco cessation.^25^^–^^28^ Recent nationally representative data indicate that the majority of current tobacco users in Japan wish to quit or reduce their tobacco use.^30^ Given this, cessation advice is crucial for all patients, regardless of their reason for seeking medical care. Since smoking cessation is a multi-stage process that typically begins with a quit attempt followed by sustained abstinence,^31^ healthcare providers are well-positioned to use every patient encounter as an opportunity for tobacco use screening, cessation counseling, and treatment.

Our findings also revealed a lower quit attempt rate among exclusive HTP users, despite similar rates of receiving cessation advice between exclusive HTP users and exclusive combustible tobacco smokers. This may be due to the perception of HTPs as a smoking cessation aid for transitioning away from combustible tobacco,^9^^,^^32^ although previous studies suggest that HTP use could hinder cessation and even lead to smoking relapse.^9^^,^^33^ Additionally, HTPs are often considered more socially acceptable than combustible tobacco,^3^ which may reduce motivation to quit their use. Both healthcare providers and patients should recognize that HTPs are not harmless and may complicate cessation efforts.^34^^–^^37^ The ultimate goal should be to achieve cessation of all forms of tobacco use.

The present study has several limitations. First, participants were recruited from an opt-in, non-probability, internet-based panel; therefore, the sample is not strictly nationally representative. To approximate the national population, recruitment used quota sampling on key demographics and all analyses applied post-stratification weights to align estimates with national demographic distributions; nonetheless, selection bias may remain, and individuals with limited internet access or literacy may be underrepresented (although 86.2% of the Japanese population had internet access in 2023).^38^ Second, the self-reported nature of the study may have introduced recall bias and inaccuracies in reporting. Third, although we adjusted for current receipt of medical care and conducted motivation-stratified sensitivity analyses, residual confounding may persist. For example, we lacked data on care indication/acuity (eg, acute tobacco-related conditions) that could influence both receipt of advice and the likelihood of attempting to quit. In addition, we were underpowered to model provider-type differences; thus, confounding by provider type cannot be ruled out and should be examined in future studies with richer clinical detail. Finally, due to the cross-sectional design, we could not establish a causal relationship between receiving cessation advice from healthcare providers and making a quit attempt. Future research using longitudinal data is needed to examine the long-term impact of healthcare providers’ advice on tobacco users’ behaviors, particularly as the landscape of tobacco use and cessation becomes increasingly complex with the emergence of HTPs.

### Conclusion

In summary, our study highlights the low rates of tobacco cessation advice provided by healthcare providers in Japan, despite its strong association with quit attempts. Given the evolving landscape of tobacco use, particularly the widespread use of HTPs, a comprehensive approach is needed to address cessation challenges. Strengthening the involvement of all healthcare providers in tobacco cessation counseling through policy initiatives, training, and system-level interventions will be essential.

## SUPPLEMENTARY MATERIAL

eTable 1. Respondent distributions overall, among tobacco users, and among tobacco users currently receiving medical care, JASTIS 2024

eTable 2. Quit attempts and association with receipt of cessation advice from healthcare providers among tobacco users currently receiving medical care, stratified by quit motivation
