# Supplementary material for: Healthcare Providers’ Advice for Tobacco Cessation and Its Association With Quit Attempts: A Cross-sectional Study in Japan, 2024
Source: J Epidemiol. 2026 Sep 5;36(9):273–80. doi: 10.2188/jea.JE20250277 (PMC13447038; doi:10.2188/jea.JE20250277)
Supplement: Supplementary file 1 [file je-36-273-s001.pdf]

eTable 1. Respondent distributions overall, among tobacco users, and among tobacco users currently receiving medical care, JASTIS 2024

	Overall (n=27,374)	Tobacco users (n=6,361)	Tobacco users currently receiving medical care (n=2,779)
	n (weighted %)	n (weighted %)	n (weighted %)
Total	27,374 (100.0)	6,361 (100.0)	2,779 (100.0)
Tobacco product type			
Non-user	21,013 (75.8)	-	-
Combustible tobacco only	3,024 (11.3)	3,024 (46.6)	1,338 (49.3)
HTP only	1,352 (5.6)	1,352 (23.3)	516 (20.4)
Dual use	1,985 (7.3)	1,985 (30.1)	925 (30.4)
Sex			
Female	13,891 (51.0)	1,947 (29.4)	793 (27.0)
Male	13,483 (49.0)	4,414 (70.6)	1,986 (73.0)
Age, years			
16–19	521 (1.9)	42 (0.6)	25 (1.1)
20–29	5,043 (18.5)	1,145 (16.7)	397 (12.7)
30–39	4,825 (17.7)	1,024 (19.4)	318 (13.7)
40–49	5,037 (18.5)	1,341 (22.9)	497 (18.8)
50–59	4,621 (16.9)	1,300 (18.8)	596 (19.4)
≥60	7,327 (26.5)	1,509 (21.6)	946 (34.3)
Education			
High school or below	7,241 (54.1)	1,953 (59.3)	861 (60.2)
Beyond high school	19,867 (45.9)	4,350 (40.7)	1,890 (39.8)
Marital status			
Single	9,293 (29.7)	2,066 (26.6)	740 (20.9)
Married/cohabiting	15,788 (61.7)	3,641 (64.2)	1,692 (67.9)
Separated/divorced/widowed	2,293 (8.6)	654 (9.2)	347 (11.3)
Urbanization			
Urban (DID)	20,301 (60.8)	4,731 (60.0)	2,044 (59.9)
Rural (non-DID)	7,073 (39.2)	1,630 (40.0)	735 (40.1)
Alcohol use			
Non-current/never	12,186 (46.9)	1,937 (31.9)	796 (29.8)
Current	15,188 (53.1)	4,424 (68.1)	1,983 (70.2)
Currently receiving medical care			
No	16,783 (61.3)	3,582 (56.9)	-
Yes	10,591 (38.7)	2,779 (43.1)	-
Quit intention			
No	-	1,818 (28.7)	707 (26.1)
Yes	-	4,543 (71.3)	2,072 (71.2)

DID, Densely Inhabited District; HTP, heated tobacco product; JASTIS, Japan Society and New Tobacco Internet Survey.
Note: Values are unweighted counts (n) and weighted percentages. Percentages are post-stratification weighted to align with the Japanese population's demographic distributions. "Currently receiving medical care" serves as a proxy for recent visits to hospitals, clinics, or dental clinics. Combustible tobacco products include cigarettes, roll-your-own cigarettes, little cigars, pipes, and water pipes. Heated tobacco products (HTPs) include Ploom Tech, Ploom S, Ploom X, IQOS, glo, and lil HYBRID.

eTable 2. Quit attempts and association with receipt of cessation advice from healthcare providers among tobacco users currently receiving medical care, stratified by quit motivation

Characteristics	Quit motivation – No (n=707)		Quit motivation – Yes (n=2,072)	
	Weighted % (95% CI)	APR (95% CI)	Weighted % (95% CI)	APR (95% CI)
Total	12.8 (8.0–17.6)	–	35.9 (32.5–39.3)	–
Receipt of cessation advice				
No advice	9.6 (5.0–14.3)	Ref.	32.7 (28.9–36.5)	Ref.
Yes (from one professional)	24.3 (6.9–41.7)	1.15 (0.60–2.19)	45.8 (37.3–54.3)	1.29 (1.05–1.60)
Yes (from two or more professionals)	57.5 (22.4–92.5)	2.36 (1.12–4.98)	58.4 (42.6–74.1)	1.48 (1.08–2.01)
Tobacco product type				
Combustible tobacco only	5.2 (2.2–8.2)	Ref.	39.4 (34.3–44.5)	Ref.
HTP only	15.2 (1.6–28.8)	2.22 (0.68–7.27)	18.0 (12.1–23.8)	0.45 (0.32–0.63)
Dual use	25.6 (15.3–36.0)	2.05 (1.05–4.02)	44.2 (38.0–50.3)	0.92 (0.75–1.13)
Sex				
Female	9.8 (2.1–17.6)	Ref.	34.9 (28.4–41.3)	Ref.
Male	13.6 (7.9–19.3)	1.38 (0.67–2.83)	36.4 (32.4–40.4)	1.03 (0.85–1.26)
Age, years				
16–19	83.1 (56.2–110.0)	3.46 (1.31–9.13)	17.0 (0.5–33.6)	0.58 (0.17–1.94)
20–29	35.0 (13.8–56.1)	Ref.	50.1 (40.0–60.1)	Ref.
30–39	10.3 (2.7–17.9)	0.32 (0.15–0.69)	45.2 (35.6–54.9)	0.97 (0.71–1.31)
40–49	10.8 (3.6–18)	0.45 (0.19–1.07)	31.7 (24.7–38.7)	0.75 (0.55–1.04)
50–59	4.0 (1.2–6.9)	0.19 (0.07–0.49)	28.0 (21.7–34.4)	0.66 (0.47–0.94)
≥60	5.2 (0.8–9.7)	0.27 (0.11–0.69)	33.7 (27.7–39.8)	0.75 (0.54–1.03)
Education				
High school or below	10.7 (3.7–17.7)	0.46 (0.26–0.82)	35.0 (29.8–40.2)	0.98 (0.83–1.16)
Beyond high school	16.7 (11.3–22.0)	Ref.	38.1 (34.5–41.7)	Ref.
Marital status				
Single	25.3 (11.6–39.0)	1.23 (0.60–2.52)	42.6 (35.2–50.0)	1.08 (0.86–1.35)
Married/cohabiting	8.5 (4.9–12.1)	Ref.	34.3 (30.3–38.4)	Ref.
Separated/divorced/widowed	6.2 (0.2–12.3)	1.09 (0.41–2.90)	34.8 (24.1–45.5)	1.06 (0.78–1.45)
Urbanization				
Urban (DID)	14.5 (7.6–21.5)	Ref.	37.1 (33.0–41.3)	Ref.
Rural (non-DID)	10.0 (4.8–15.1)	1.10 (0.61–2.00)	34.2 (28.5–39.9)	0.91 (0.75–1.10)
Alcohol use				
Non-current/never	9.3 (3.4–15.3)	Ref.	31.0 (24.9–37.2)	Ref.
Current	14.2 (8.0–20.5)	1.14 (0.49–2.65)	38.0 (34.0–42.1)	1.16 (0.94–1.43)

APR, adjusted prevalence ratio; CI, confidence interval; DID, Densely Inhabited District; HTP, heated tobacco product; Ref., reference.
Note: Receipt of healthcare providers' advice and quit attempts were both assessed over the past year. Data were weighted to address the selectivity of the internet-based sample using a nationally representative sample of Japanese population as the reference. "Currently receiving medical care" serves as a proxy for recent visits to hospitals, clinics, or dental clinics. Combustible tobacco products include cigarettes, roll-your-own cigarettes, little cigars, pipes, and water pipes. Heated tobacco products (HTPs) include Ploom Tech, Ploom S, Ploom X, IQOS, glo, and lil HYBRID. Quit motivation was defined from product-specific items asking whether respondents were interested in quitting HTPs or combustible tobacco within 1 month / within 6 months / sometime (coded yes) versus not interested (coded no).
